# Perianal herpes ulceration in a HIV positive person

**DOI:** 10.11604/pamj.2020.35.7.19247

**Published:** 2020-01-09

**Authors:** Wafaa Bennane, Youness Chakir

**Affiliations:** 1 Infectious Disease Service, Chu Ibn Rochd, Casablanca, Morocco; 2 Laboratory Sexual Health, Faculty of Medicine, Casablanca, Morocco

**Keywords:** Perianal ulceration, HIV, herpes

## Image in medicine

Mr ZA, aged 56, from the Ivory Coast, followed for HIV revealed by cutaneous kaposi with uro-genital location in 2016, under treatment, consulted for perianal ulceration evolving since 1 year. On examination, the patient was febrile, cachectic, in poor general condition, with maculopapular lesions angiomatous in the lower limbs, external genital organs without involvement of the oral mucosa. The clinical examination found a perianal ulcer wide of 12cm long axis, painful, itchy with sharp edges with extension to the external genitalia. The rectal examination is painful, the fingerstall returns stained with blood, with extension of the lesion in endo-anal. Histopathological and cytological examination was in favor of a herpes infection, the culture confirmed the herpes infection.The patient had a CD4 count of 159 elements/mm^3^, and a viral load of 71 copies/ml, negative syphilitic serology, negative CMV PCR and normal fundus. In addition, the patient had a normochromic normocytic anemia at 5.9 g/dl, thrombocytopenia at 19000 elements/mm3 and white blood cells at 630 elements/mm^3^, non-conclusive myelogram, CRP increased to 234 mg/l, LDH at 1672 IU/l, Ferritinemia at 9000 μg/dl and TG at 2.5 μg/l, a hypofibrinogenemia at 1 μl. The patient received a bolus of corticosteroid therapy with suspicion of macrophage activation syndrome, and the ulceration was treated with acyclovir 10mg/kg/h with a good clinical course.

**Figure 1:**
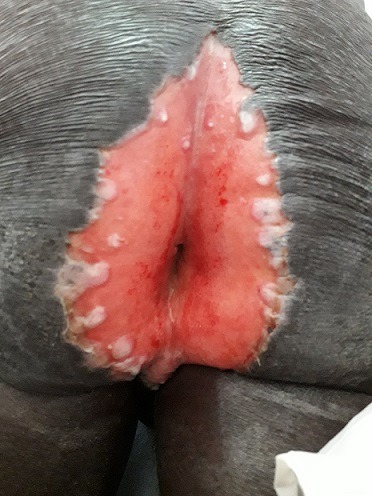
perianal herpes ulceration in a HIV positive person

